# Boosted Regeneration and Reduced Denervated Muscle Atrophy by NeuroHeal in a Pre-clinical Model of Lumbar Root Avulsion with Delayed Reimplantation

**DOI:** 10.1038/s41598-017-11086-3

**Published:** 2017-09-20

**Authors:** David Romeo-Guitart, Joaquim Forés, Xavier Navarro, Caty Casas

**Affiliations:** 1 Institut de Neurociències (INc) and Department of Cell Biology, Physiology and Immunology, Universitat Autònoma de Barcelona (UAB), Bellaterra, Barcelona, Spain; 2 Hand and Peripheral Nerve Unit, Hospital Clínic i Provincial, Universitat de Barcelona, Barcelona, Spain

## Abstract

The “gold standard” treatment of patients with spinal root injuries consists of delayed surgical reconnection of nerves. The sooner, the better, but problems such as injury-induced motor neuronal death and muscle atrophy due to long-term denervation mean that normal movement is not restored. Herein we describe a preclinical model of root avulsion with delayed reimplantation of lumbar roots that was used to establish a new adjuvant pharmacological treatment. Chronic treatment (up to 6 months) with NeuroHeal, a new combination drug therapy identified using a systems biology approach, exerted long-lasting neuroprotection, reduced gliosis and matrix proteoglycan content, accelerated nerve regeneration by activating the AKT pathway, promoted the formation of functional neuromuscular junctions, and reduced denervation-induced muscular atrophy. Thus, NeuroHeal is a promising treatment for spinal nerve root injuries and axonal regeneration after trauma.

## Introduction

Traumatic injuries to the spinal roots and brachial or lumbar nerve plexus usually result in permanent loss of motor and sensory functions in the affected members. Advanced microsurgical interventions by neurotization and nerve transfer^1^ or, in some cases, by direct nerve reimplantation of injured roots has been shown to allow some functional recovery in cases with brachial plexus avulsion^2–4^. Although the outcome is dependent on age of the patient and delay of intervention, generally protective sensation is recovered, but there is considerable muscle atrophy and poor motor functional recovery^5^.

Proximal nerve injuries result in three main problems. First, the rupture of the ventral roots results in a progressive retrograde neurodegeneration of axotomized motoneurons (MN)^6–8^ that compromises motor functional recovery. Second, the long distances that injured motor axons have to regrow to reach the muscles of denervated limb makes the chances for reinnervation very limited^9,10^. Third, there is muscle atrophy due to long-term denervation^2^. Therefore, any envisaged therapeutic strategy must consider these aspects as a whole.

In animal models of root avulsion (root avulsion, RA), immediate reimplantation of the avulsed roots increases MN survival and allows some reinnervation of limb muscles although with limited functional recovery that is worse in lumbar than in cervical root injuries, likely due to the differences in length^11–14^. To prevent MN atrophy and enhance axonal outgrowth and fiber density along roots, several experimental studies have induced expression of neurotrophic factors by gene therapy with some positive effects; however, in areas with continuously elevated levels of neurotrophic factor the axons remain trapped and do not grow to distal targets^15,16^. Grafting of mesenchymal stem cells into the injured spinal cord segments have shown some benefit^17,18^. Drugs, such as riluzole^13^, lithium^19^, and intracellular sigma peptide (ISP, a mimetic of the proteoglycan receptor PTPσ)^20^, have been tested only after immediate reimplantation of the avulsed roots and so have limited translational potential.

In order to bring efficient therapeutic strategies to the clinic, we developed a preclinical model based on RA plus delayed surgical reimplantation of lumbar roots to test a new promising drug combination called NeuroHeal^7^. NeuroHeal was discovered using unbiased proteomic data from two models that represented pure regenerative and pure neurodegenerative conditions after nerve or RA injuries, respectively. The data served to build bona fide state-specific molecular maps and mathematical models of this human biological system that allowed us to screen databases of drugs to identify putatively neuroprotective combinations. NeuroHeal is a combination of FDA-approved drugs. Results presented here evaluating NeuroHeal in a preclinical model demonstrate the promise of this coadjuvant agent for the clinical treatment of root and plexus injuries.

## Results

### NeuroHeal promotes motoneuron survival and reduction of glial scars in a preclinical model of RA with delayed repair of lumbar roots

**Figure 1:**
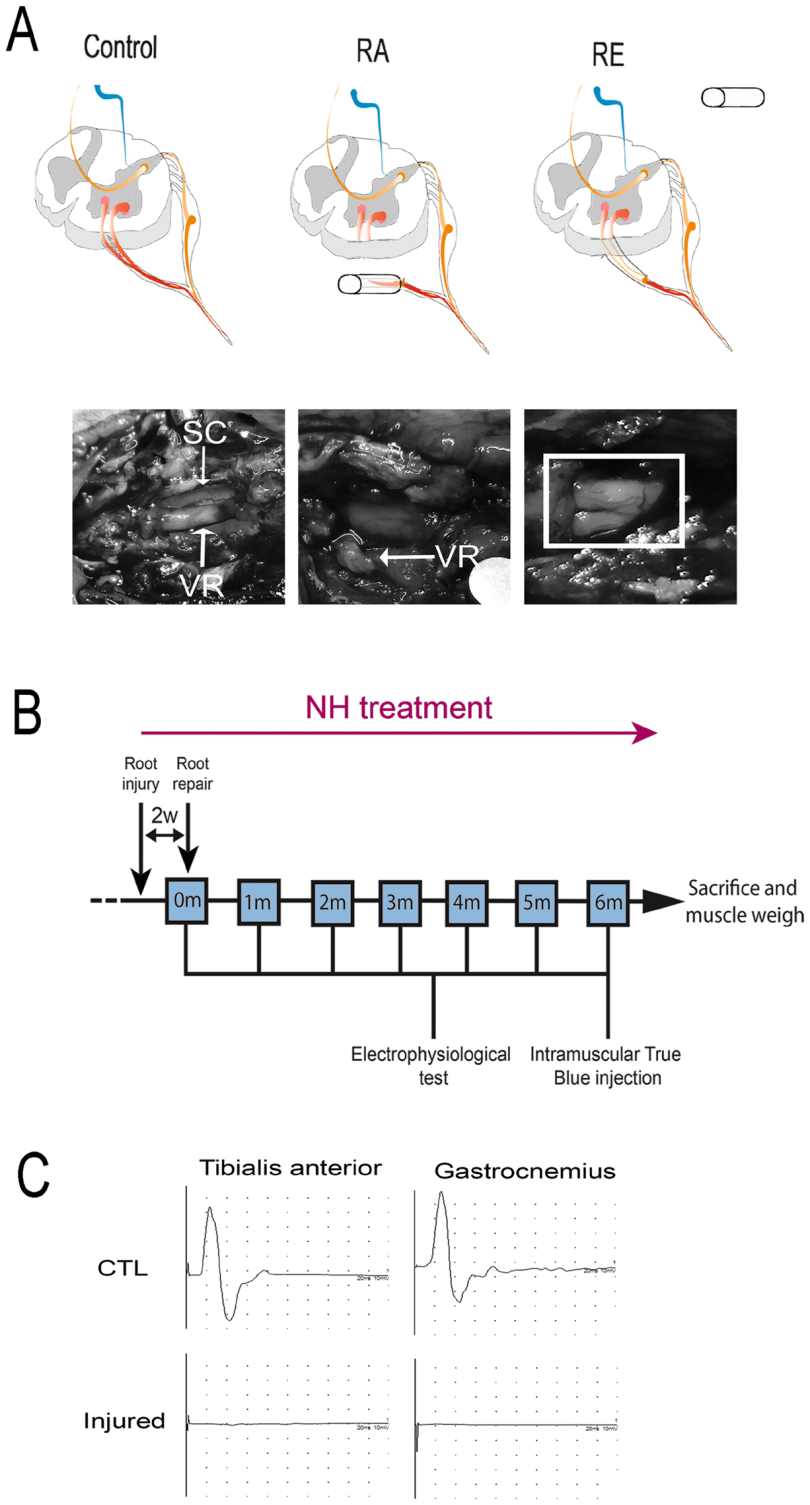
*In vivo* preclinical model of RA injury with delayed reimplantation of lumbar roots and experimental design. **(A)** Schematic of the procedure. Spinal cord with the nerve roots from control, root-avulsed (RA), and reimplanted (RE) animals. The L3-L6 spinal roots were detached and inserted into a silicone tube after RA injury. At 14 dpi, the tube was removed, and the injured spinal roots were re-inserted onto the spinal cord at the same lumbar level. Photographs showing the spinal cord (SC) and ventral roots (VR) inside the tube (left), root appearance after removing the tube (middle) and once roots were apposed underneath the spinal cord (right), during reimplantation surgery at 14 dpi. **(B)** Workflow of the experimental design. NH treatment was administered from the day of injury dissolved in the drinking water refreshed every three days for 6 months. Fourteen days post avulsion, a group of animals were reimplanted. A week later, some animals were sacrificed to evaluate MN survival (short-term period: 3 weeks post-RA injury or 1 week post-RE). The rest of animals were evaluated once per month with electrophysiological tests. One week before the end of the 6-month follow up period, we intramuscularly injected True Blue retrotracer at the tibialis anterior (TA) and the gastrocnemius (GA). **(C)** Electrophysiological CMAP recordings of control animals (CTL) and animals after RA and before RE (Injured).

We performed RA by traction of the L3 to L6 ventral spinal roots followed by reimplantation (RE) at 14 days post-injury (dpi). To facilitate root handling, we maintained the transected roots in a small silicone tube during the 14-day period prior to reimplantation (Fig. 1A). After reimplantation, we evaluated the animals using electrophysiological tests over 6 months. NeuroHeal-treated animals were given the combination therapy in drinking water from the day of injury. Although we observed, rats were drinking the half during the first 2 days, they continue to drink regularly during the 6 months that the treatment last. At the end of the follow-up period, we injected True Blue retrotracer to label regenerated MNs (Fig. 1B). At 14 days after RA none of the animals had compound muscle action potential (CMAP) responses in the tested muscles, indicating complete loss of motor function and confirming the effectiveness of the surgical approach (Fig. 1C).

**Figure 2:**
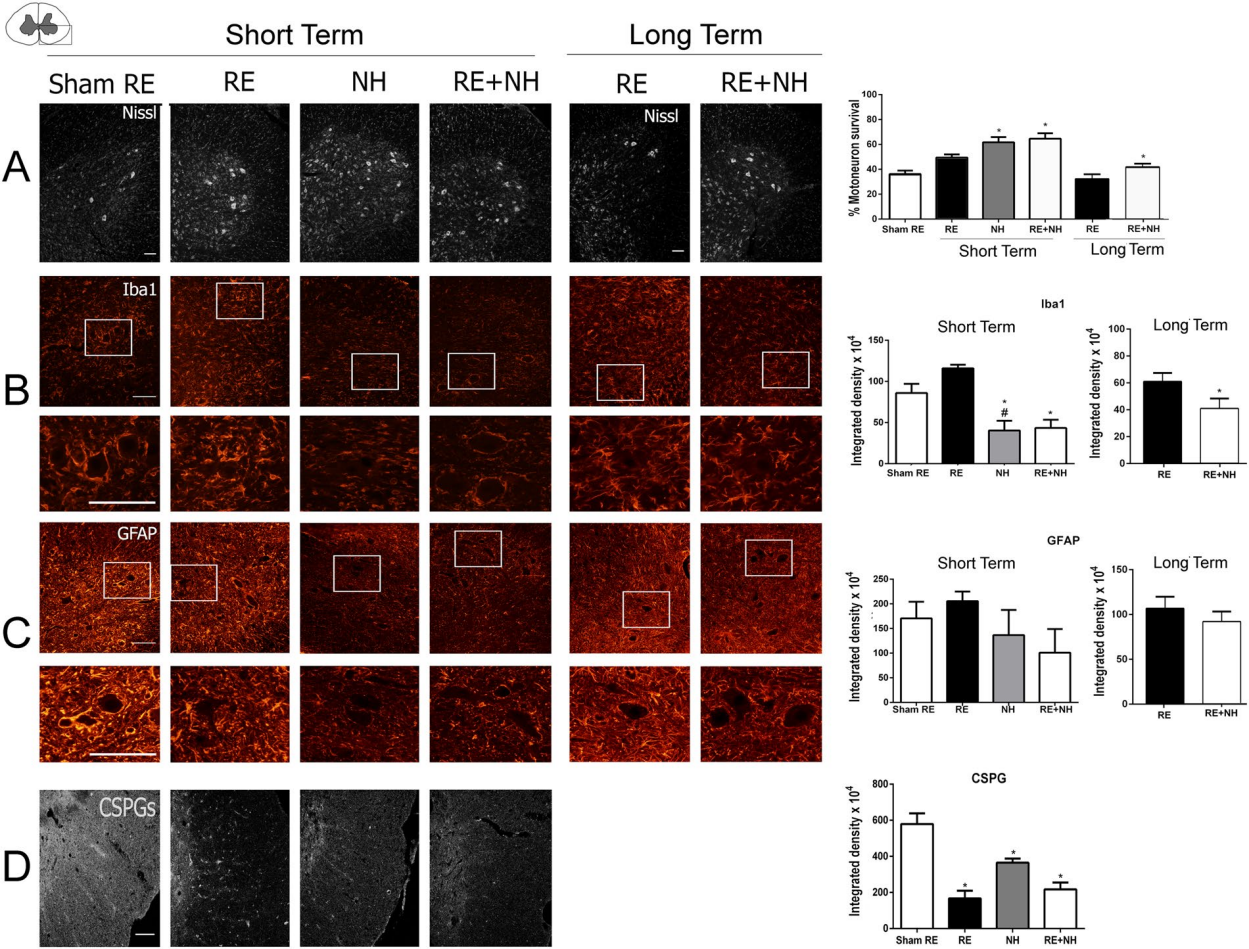
NH treatment has short- and long-term neuroprotective effects in the preclinical model. (**A**) ***Left***, Representative microphotographs of ventral horns of root-avulsed spinal cords stained with fluorescent Nissl, which specifically labels MNs, at 21 dpi post RA and 1 week post RE (short term) or at 6 month post RE (long term) from RA injured untreated animals (sham RE) or NeuroHeal-treated animals (NH and RE + NH). ***Right***, Bar graphs showing the average percentage of surviving MNs ± SEM on the injured side with respect to the contralateral side. **(B**–**D)** ***Left***, Representative microphotographs of ventral horns from injured animals with labeled microglia (Iba1), astrocytes (GFAP), at low (top) and high magnification (below), or chondroitin sulfate proteoglycans (CSPGs), respectively. ***Right***, Histograms of the averaged immunoreactivity in the fixed region of interest at the ventral horn from different groups (n = 4 for each group, ANOVA, post hoc Bonferroni, *p < 0.05 RE + NH vs. Sham RE or RE). Scale bar = 100 µm.

Neuronal survival was assessed as the ipsilateral to contralateral ratio of MNs located in the lamina IX of the ventral horn (Fig. 2A). In the sham RE group, animals were subjected to RA and then to mock surgery at 14 dpi. As expected, sham animals presented with a significant drop in the number of avulsed MNs in the ipsilateral side at 21 days post-RA relative to the contralateral side (Fig. 2A). The animals subjected to RA and to RE (group RE) had increased MN survival compared to unrepaired rats but the difference was not significant (RE 49.44% ± 2.48; sham RE 36.2% ± 2.73). In contrast, RA avulsed animals subjected to either NeuroHeal treatment (NH group) or to reimplantation in addition to NeuroHeal treatment (RE + NH group) showed an increased proportion of surviving MNs of 61.71% and 64.62% respectively compared to sham RE group at 1 week after RE (Fig. 2A, short term: 3 weeks after RA or one week after RE in already RA injured animals). At 6 months, the animals treated with NeuroHeal had higher number of surviving MNs than those in the RE group, indicating a beneficial long-lasting effect of the treatment (Fig. 2A, long term).

Immunoreactivity against Iba 1 and GFAP was used to analyze the degree of microglial and astroglial reactivity after lesion, respectively. At 21 days post RA, there was similar glial reactivity in both the sham RE and RE groups (Fig. 2B,C). In contrast, Iba1 immunoreactivity was significantly reduced in all the animals treated with NeuroHeal at both early and late time points (Fig. 2B); however, there was no significant reduction of astroglial reactivity at any time (Fig. 2C; short term: 170.58% ± 33.65 in sham RE, 98.39 ± 47.96% in NH, and 100.97 ± 47.78% in NH + RE groups; and long term: 106.52 ± 13.27% in RE and 91.75 ± 11.52% in NH + RE). We did observed a significant reduction in chondroitin sulfate proteoglycan (CSPG) content, a matrix component of the glial scar reaction, in all the treated groups with respect to the untreated sham group at the time of its formation by 7 days after RE **(**Fig. 2D). These results suggested that NeuroHeal treatment exerted long lasting neuroprotection when begun after delayed surgical repair of avulsed lumbar roots.

### NeuroHeal treatment enhances regeneration of motoneurons

**Figure 3:**
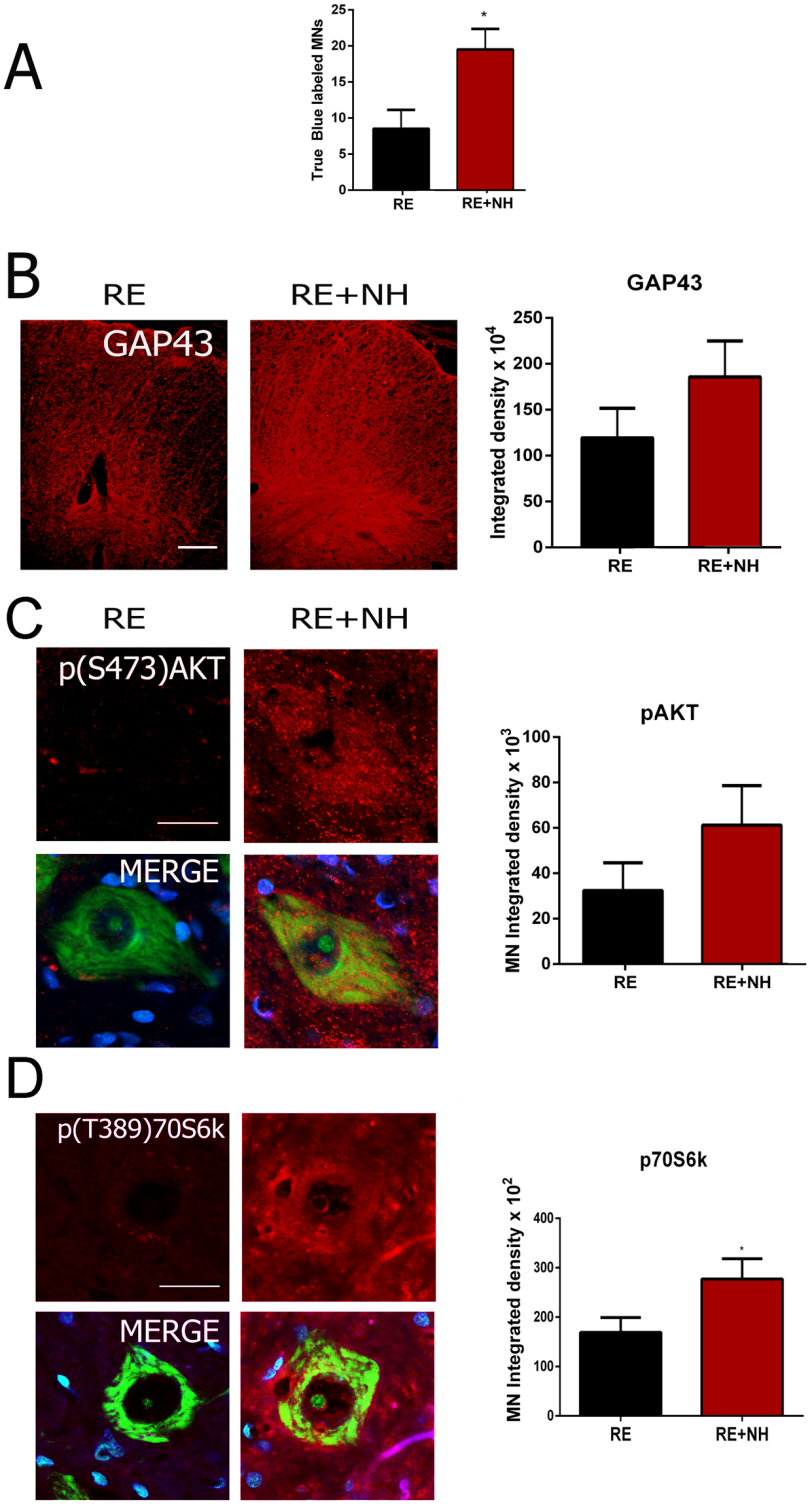
Molecular markers of regeneration are modulated by NH treatment. **(A)** Histogram showing the average number (±SEM) of MNs labeled after intramuscular injection of True Blue into the TA and GA on the injured side. The number of positive MNs were counted in the L4-L5 segments of the spinal cords from animals at 6 months post RE for animals in RE group (not treated) or NH-treated groups (RE + NH). **(B)** ***Left***, Representative microphotographs of GAP43 immunolabeling of neuronal processes at the white matter ventral horns from injured animals. ***Right***, Bar graph of the mean immunolabeling intensity for GAP43 in a region inside the white matter from injured spinal cords. **(C**,**D)** ***Left***, Confocal images of MNs immunolabeled for (**C**) phosphorylated AKT at S-473 and D) p70S6K at T-389 and counterstained with Fluoro Nissl Green and DAPI (blue) in injured spinal cord at 6 months post RE. ***Right***, Histograms of the mean of the immunofluorescence intensity for each marker inside the cytoplasm of injured MNs (n = 4 animals, t-test, *p < 0.05 RE + NH vs. RE). Scale bar = 100 µm in **B**; 25 µm in **C** and **D**.

At 6 months after RE, by retrograde labeling by injection of True Blue into the tibialis anterior and gastrocnemius, we observed 2 fold more regenerated MNs in NeuroHeal-treated reimplanted animals compared to those reimplanted but untreated (Fig. 3A). We then evaluated the presence of pro-regenerative molecular marker GAP43 and activation of putative NeuroHeal target such as AKT^7^. NeuroHeal treatment increased GAP43 and phosphorylated AKT levels, suggesting activation of pro-regenerative programs (Fig. 3B,C). Expression of phosphorylated p70S6k (Thr 389), a downstream AKT/mTOR target, was markedly increased within MNs in the NeuroHeal-treated RE animals compared with untreated animals (Fig. 3D).

### Long-term NeuroHeal treatment accelerates nerve regeneration and recovery

**Figure 4:**
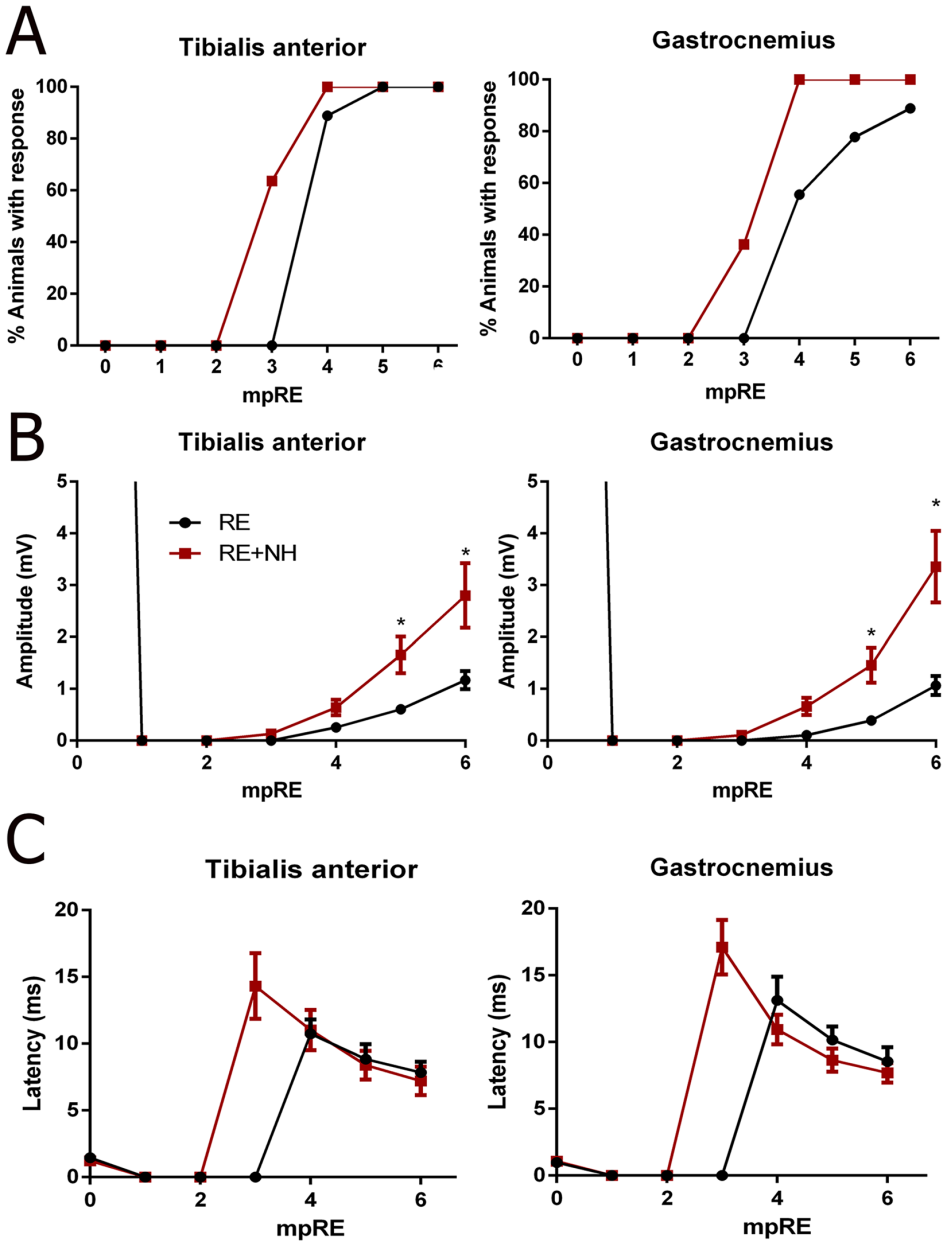
Nerve regeneration and CMAP amplitudes are enhanced by NH treatment. **(A**) Graphs summarizing the percentages of animals in RE group (not treated) or NH-treated groups (RE + NH) with electrophysiological signals of muscle reinnervation in TA and GA muscles at indicated times after RE. **(B)** Mean amplitudes (±SEM) and **(C)** time latency values of CMAP recordings obtained during follow-up post RE from TA and GA muscles (n = 9–11, ANOVA, post hoc Bonferroni, *p < 0.05 RE + NH vs. RE).

In addition to confirming neuroprotection exerted by NeuroHeal, we investigated whether NeuroHeal treatment improved motor recovery in our model by using electrophysiological tests monthly. CMAPs were observed in the NeuroHeal-treated group between 2 and 3 months after RE. In contrast, no responses were observed in the untreated group until 4 months after RE (Fig. 4A), indicating that NeuroHeal treatment accelerated axonal growth by at least 4 weeks (p = 0.0276 in TA; p = 0.0053 from GA). By 4 months, all the NeuroHeal-treated rats presented electrophysiological evidence of reinnervation in both muscles, whereas in 11% of untreated animals there was no evidence of reinnervation at 6 months. Moreover, the NeuroHeal-treated group had significantly higher CMAP amplitudes compared to the untreated group at 5, and 6 months (p < 0.05, Fig. 4B). No significant differences in the CMAP latencies were observed (Fig. 4C).

**Figure 5:**
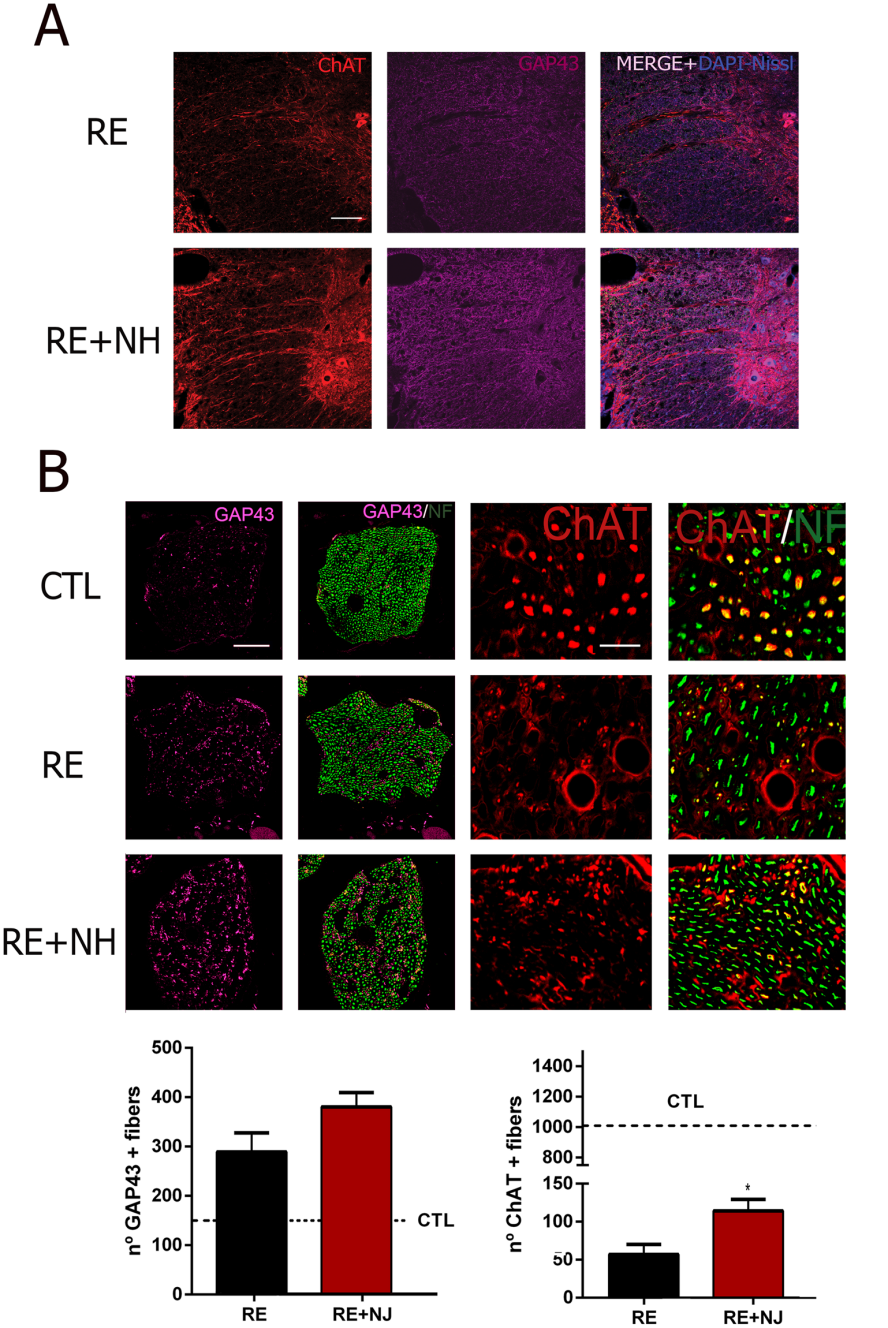
Regeneration of motor axons increased by NH treatment. **(A)** White matter of the spinal cord immunostained against ChAT (red), GAP43 (purple), and counterstained with DAPI (blue, for nuclei) and Fluoro Nissl Blue (for MNs) RE and NH-treated (RE + NH) animals at 6 months post RE. **(B)** ***Top***, Microphotographs showing co-localization of GAP43 (purple) or ChAT (red) with neurofilament NF200 (NF, green) at mid-level of sciatic nerves obtained from controlateral (CTL), RE, and RE + NH groups at 6 months post RE. ***Bottom***, Bar graphs showing the average (±SEM) of the number of immunohistochemically detected GAP43^+^ (left) and ChAT^+^ (right) fibers at midlevel of sciatic nerve. Dotted lines indicate control value means for GAP43 and ChAT, respectively (n = 3 for CTL group and n = 4 for RE and RE + NH; t-test, *p < 0.05 RE + NH vs. RE). Scale bar = 100 µm for **A** and for GAP43/NF in **B**, and 25 µm for ChAT/NF in **B**.

Analysis of spinal cords at L4-L6 showed abundant acetyl cholinesterase-positive (ChAT^+^) motor fibers along the ventral horn in NeuroHeal-treated animals at 6 months after RE (Fig. 5A). Accordingly, in the sciatic nerve there were significantly higher numbers of ChAT^+^ motor axons from surviving MNs (up to 11.30% of the total L4-L6 pool) and a trend to a higher number of regenerative GAP43^+^ fibers entering the reimplanted root in NH-treated animals compared to those in the RE group (Fig. 5B).

### Reduced muscle atrophy and increased functional endplates observed in NH-treated rats

**Figure 6:**
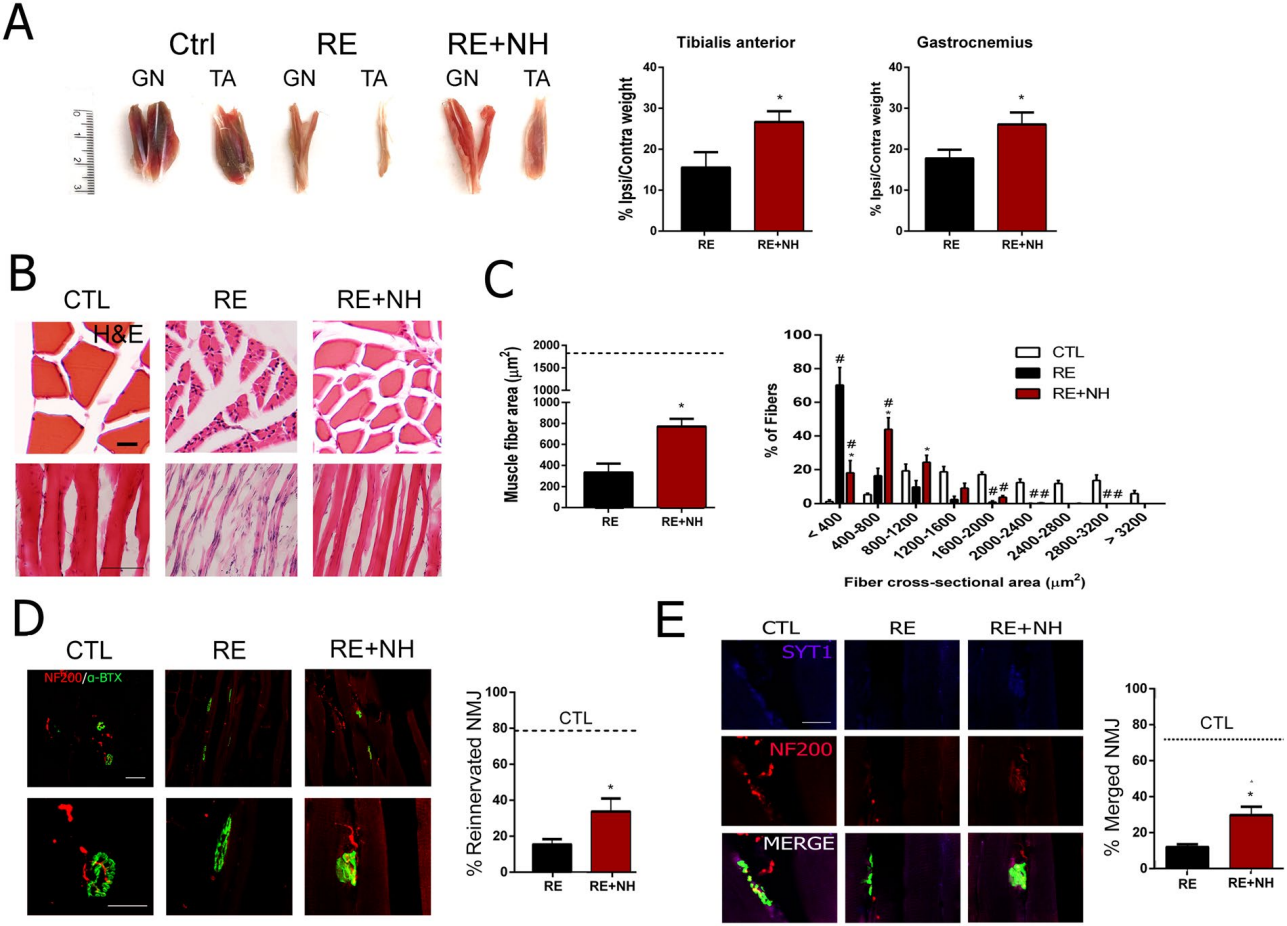
Muscle atrophy is reduced and numbers of functional endplates are increased by NH treatment. (**A)** ***Left***, Representative photographs of GA and TA muscles from contralateral regions of RE rats (CTL) and ipsilateral from untreated (RE) and NH-treated (NH + RE) rats at 6 months post RE. ***Right***, Histograms of weight ratio between ipsilateral and contralateral TA and GA muscles at 6 months post RE (n = 7–8; t-test, *p < 0.05 RE + NH vs. RE). **(B)** Representative microphotographs of cross (upper panel) and longitudinal (lower panel) TA and GA muscle sections with H&E staining from CTL, RE, and RE + NH groups at 6 months post RE. **(C)** ***Left***, Means (±SEM) of the fiber cross-sectional areas (µm^2^) from GA muscles of indicated groups at 6 months post RE. Dotted line indicates the mean obtained from the CTL muscles (n = 4 per group; t-test, *p < 0.05 RE + NH vs. RE). ***Right***, Histogram of the cross-sectional area distribution of fibers in GA. Each interval of the distribution contains ranges of 400 µm^2^ between the extremes (n = 4 per group), ANOVA, post hoc Bonferroni *p < 0.05 RE + NH vs. RE). **(D)** ***Left***, Representative confocal images of GA transversal sections stained for NF200 (NF, red) and FITC-conjugated α-bungarotoxin (α-btx, green) from contralateral side of RE rats (CTL), ipsilateral side of RE rats, and NH-treated animals (RE + NH) at 6 months post RE. ***Right***, Quantification of the percentage of reinnervated NMJs (red and green overlapping signal) analyzed with ImageJ software in RE and RE + NH groups compared to CTL (dotted line; n = 3–4; t-test, *p < 0.05 RE + NH vs. RE). **(E)** Representative microphotographs of SYT1 immunolabeling (magenta) and NF co-localization (red) in α-btx (green) neuromuscular junctions (NMJs). Scale bars = 20 µm in B (bottom panel) and E; 25 µm in D (bottom panel); 50 µm in D (upper panel); and 100 µm in B (upper panel).

We also evaluated recovery from muscle atrophy due to denervation. Muscle weight is a sensitive measure of muscle atrophy. We harvested and weighted the gastrocnemius and tibialis anterior muscles of both ipsi- and contralateral sides from the NH-treated and untreated animals. We found an average 83% reduction in the weight of both TA and GA ipsilateral muscles compared to contralateral muscles in the RE group consistent with persistent long-term denervation-induced atrophy. In contrast, NH treatment reduced muscle atrophy compared to untreated rats with muscle reduction of about 72% with respect to the contralateral side (p < 0.05, Fig. 6A). Long-term denervated muscle fibers have cells with shrunken cytoplasms, show signs of fibrosis, and cluster into groups^21,22^. In addition, the more widespread the denervation, the greater the percentage of small muscle fibers^23^. To evaluate muscle fibers, we analyzed muscle sections histologically. The RE group of animals had fibers with small cross-sectional areas and numerous fibroblast nuclei within the endomysium suggesting fibrosis (Fig. 6B). In contrast, NH treatment fibers presented clear myocyte nuclei and no apparent fibrosis. In addition, in the RE group, fibers were contracted by 82% relative to contralateral regions of RE rats, but animals treated with NH had fibers contracted only 58% relative to controls (p < 0.001, Fig. 6c). In muscle sections from the RE group we observed clustering of small fibers; most were smaller than 400 µm^2^. NH treatment resulted in a more distributed mean cross-sectional area with the most abundant fibers those between 400 and 800 µm^2^ (Fig. 6C). These observations are consistent with the extension of reinnervated motor endplates in the muscles.

We then analyzed the folds of neuromuscular junctions (NMJs) of GA muscle using NF-200 to stain axon terminals and FITC-conjugated α-bungarotoxin to stain acetyl-choline receptors (AchRs). There was a significantly higher percentage of reinnervated motor endplates in the NH-treated group than in the RE group (Fig. 6D). Recently it has been proposed that failure of motor recovery after long-term denervation might be due to failure in pre-synaptic function at NMJs despite physical contact^24,25^. Thus, we performed immunolabeling for syntaxin 1 (SYT1), a protein key to functional release of neurotransmitters at presynaptic terminals. We observed that this marker was present only in control and NH-treated animals and not in the RE group rats (Fig. 6E). These observations suggested that NH-treatment may promote muscle recovery and formation of functional motor endplates.

## Discussion

The findings of this work indicate that NeuroHeal treatment after root avulsion and delayed reimplantation of the lumbar roots remarkably promoted regeneration resulting in (1) long-lasting protection of motoneurons from retrograde neurodegeneration; (2) activation of a pro-regenerative profile via pAKT-mTOR signaling; (3) evidenced motor axonal regrowth with reduced gliosis formation at the transitional zone; (4) accelerated motor axonal regeneration; (5) enhanced functional reconnection of endplates, and (6) reduced long-term muscle atrophy.

The effective recovery of motor function after spinal root injuries depends on neuroprotection and axonal regeneration toward the muscle targets. In human patients, reimplanting the avulsed spinal roots into the spinal cord partially restores motor function but only if the repair procedure is performed less than 1 month after injury^2^. After brachial plexus or proximal nerve injuries the proximal muscles in the limb may recover good function, whereas distal muscles, such as intrinsic muscles of the hand, rarely regain any useful activity, although this has been described in some patients^26^.

Any therapeutic strategy must be tested in a relevant experimental model and must show significant effects on neuroprotection and nerve regeneration after long-term denervation. Here we first characterized a rat model of root avulsion with delayed reimplantation of lumbar roots. We evaluated the effects of NeuroHeal given orally in this model. One of the most remarkable effects exerted by NeuroHeal was the acceleration of axonal regeneration. Very few strategies have demonstrated this property with exceptions of the treatment with FK506^27^ and the use of brief electrical stimulation (ES)^28,29^. Although FK506 accelerated axonal regeneration by still unknown mechanisms, its secondary effects as immunosuppressant preclude clinical use. FK506 and geldanamycin, which also accelerates axonal regeneration, do not reduce muscle atrophy as NH does^30^. ES to the nerve is being used clinically^31^. Results obtained by ES and by NH treatment are comparable since both advanced axonal regeneration at least 4 weeks. However, nerve ES has not been shown to reduce muscle atrophy. NH may act specifically to preserve muscle integrity during the time of denervation. Recently, failure of motor recovery after long-term denervation has been demonstrated to be due to failure in pre-synaptic function of endplates despite physical contact^24,25^. Evidence indicates that there is an apparent mismatch in the correlation of nerve recovery and function recovery as demonstrated in a study of over 300 patients followed for 3–18 years after sciatic nerve injury^32^. Thus, the reduction in muscle atrophy that we observed here in rats treated with NH should be further investigated.

NH is a multitarget compound that was specifically designed for treatment of root avulsion injuries. Multitargeting gives the opportunity to act through several pathways in parallel to support a neuroprotective and pro-regenerative phenotype. In the present study we confirmed that one of these targets is the AKT pathway as suggested previously based on studies *in silico* ^7^. The essential role of PI3K/AKT signaling in stimulating axon regenerative processes in the adult central and peripheral nervous systems is well documented^33–36^. Activation of PI3K/AKT signaling appears to result from ablation of its cell intrinsic antagonist, the phospholipid phosphatase PTEN^37^. The clinical use of PTEN inhibitors is not straightforward due to its anti-oncogenic properties^38^. NH offers a promising alternative since it is based on the repurposing of two already FDA-approved drugs. In addition, this multitarget aspect also may favor its concurrent action on several other critical tissues in addition to the nervous system.

Our results suggest that NH triggers specific pathways to prevent muscle degeneration. In particular, the AKT/mTOR/p70S6K pathway is known to increase protein synthesis that is necessary to maintain muscle mass^39,40^, although the role of mTOR in muscle atrophy is still controversial^41^. Another possibility is that NH activates of SIRT1^7^, a nicotinamide adenosine dinucleotide-dependent histone deacetylase that plays an essential role in regulating multiple biological processes^42^. Previous studies showed that SIRT1 overexpression induced through impeding FoxOs and NF-κB expression is capable of thwarting the loss of muscle mass, as well as inducing hypertrophy of normal muscle^43^. Indeed, SIRT1 transgenic muscle exhibits a decreased expression of the atrophy gene program^44^. Thus it would be very interesting to know whether NH prevents muscle atrophy by ameliorating related pathologies. In conclusion, we believe the treatment with NH offers interesting possibilities for treatment after nervous system trauma or upon other forms of neurological damage as in lesioned optic nerves or corticospinal tract in which central nervous system axons must regenerate to enable normal function.

## Materials and Methods

### Animals and surgical procedures

Sprague-Dawley female rats aged 12 weeks (weight at time of surgery: 250–300 g, Animal Service, *Universitat Autònoma de Barcelona*) were kept under standard conditions of light and temperature and fed with food and water ad libitum. We performed surgical procedures under anesthesia with ketamine/xylazine (100:10 mg/kg weight, i.p). For RA-injury, a midline skin incision was made, the paravertebral muscles were smoothly retracted avoiding muscle damage. Then a laminectomy of around 1 cm was made at T12-L1 level using the last rib as reference and the pia matter was opened to expose the spinal ventral roots. The L3-L6 ventral roots were detached from their entrance into the spinal cord using moderate traction with a hook, disrupting nerve integrity, as previously reported^8^. We placed the ventral roots in a silicone tube (2 mm i.d., 3 mm lenght) that was placed into the laminectomized space close to the spinal cord, avoiding damage and preventing spontaneous axon regeneration into the avulsed root. Fasciae were sutured and the skin closed with planes. Two weeks after RA injury, we anesthethized the animals, confirm denervation by electrophysiological test and proceed with the reimplantation (RE) surgery. We localized the silicone tube after blunt dissected paraventral muscles. The avulsed ventral roots were dissected back into scar-free, healthy-appearing nerve tissue, and tensionlessly were equidistantly apposed 1 mm-approximately underneath the corresponding spinal cord segment adjacent to the avulsion zone. Paravertebral muscles were opposed and pressing onto the spinal cord to avoid root misleading. In pilot studies, we had assured this procedure was enough to maintain physical connection without damaging the spinal cord in contrast of being sutured by analyzing the existence of ChAT- labeled axons in the replanted roots 14 days after reparative RE. After RE surgery, the wound was sutured by planes and disinfected with povidone iodine, and the animals were allowed to recover in a warm environment. RE-sham animals were submitted to RA-injury and re-opened two weeks after injury to remove the silicone tube. When sacrifice the animals, we visually checked that re-implanted nerve roots still remain in contact with the injured spinal cord. All procedures involving animals were approved by the Ethics Committee of *Universitat Autònoma de Barcelona*, and followed the European Community Council Directive 2010/63/EU. Groups for short-term analysis (21 dpi) were: Group Sham-RE, vehicle-treated RA-injured animals that have been just re-opened two weeks after injury; Group NH, NH-treated RA-injured animals that have been just re-opened two weeks after injury; Group RE, vehicle-treated RA-injured animals with root reimplant two weeks after injury; Group RE + NH, NH-treated RA-injured animals with root reimplant two weeks after injury. For the long-term analysis, groups were composed with RA-injured animals reimplanted at two weeks post injury treated with either vehicle (group RE) or NH (group RE + NH) for 6 months.

### Electrophysiological tests

For electrophysiological evaluation, rats were anaesthetized with ketamine/xylazine (100:10 mg/kg weight, i.p) every month after 1-month post RE surgery. The sciatic nerve was stimulated by transcutaneuous electrodes placed at the sciatic notch by single pulses (20 µs), and the CMAP was recorded by placing electrodes on the gastrocnemius and tibialis anterior. Stimulus intensity was applied gradually until reach the supramaximal stimulus, which correspond to the maximum CMAP amplitude. The evoked action potentials were displayed on a storage oscilloscope (Synergy Medelec, Viasys HealthCare) at settings appropriate to measure the amplitude from baseline to peak and the latency to the onset after every stimulus. (n = 9–11). After testing, animals were allowed to recover in a warm environment.

### Retrograde axonal tracing

To identify regenerated avulsed MNs that had reinnervated the gastrocnemius medialis and tibilais anterior muscles, we applied True Blue (Setareh Biotech) retrotracer to the muscle one week before euthanizing the animals as described elsewhere^45^. Briefly, under anesthesia with ketamine/xylazine as mentioned, we made a small cut to the skin to expose the muscle and retrotracer (6 μL) was distributed throughout the body of the muscle with a glass pipette using a Picospritzer. Pipette was maintained 10 s after injection to avoid reflux and then the animals were allowed to recover in a warm environment.

### Drug treatment

The Neuroheal mixture is composed of Acamprosate (Merck, Darmstadt, Germany) and Ribavirin (Normon, Madrid, Spain). Pills of both compounds were ground into fine powder and dissolved in drinking water at final concentration of 2.2 mM and 1 mM, respectively. Rats in the NH treatment group were given water containing drugs from the day of RA injury. Fresh drug solutions were made every 3 days.

### Tissue processing for histology

Rats were sacrified at 21 days post RA to evaluate MN survival or at 6 months after RE for analysis of nerve regeneration with dolethal (60 mg/kg, i.p.). We then transcardially perfused the animals with a saline solution containing heparin (10 U/mL), followed by 4% paraformaldehyde in 0.1 M phosphate buffer, pH 7.2, for tissue fixation. We removed L4-L5 spinal cord segments (5 mm total length), sciatic nerve from lumbar plexus to sciatic notch of the avulsed side, and tibialis anterior and gastrocnemius muscles. Spinal cord and sciatic nerve were post-fixed in the same fixative solution for 2 hours or 30 min, respectively, and cryopreserved in 30% sucrose. For muscle analysis, we weighed the ipsilateral and contralateral muscles and placed them into cryopreservation solution to avoid autofluorescence. The ratio of ipsilateral to contralateral muscle weight was calculated to assess the extent of muscle atrophy. For spinal cord analysis, we cut the samples into serial transverse sections of 20-µm thickness, obtaining 30 series with 10 sections. For sciatic nerve analysis we obtained 10 series of eight 15-µm sections. Samples were sectioned using a cryotome (Leica) and preserved at −20 °C until use. We cut gastrocnemius muscle at midlevel into 10-µm serial transverse or longitudinal sections obtaining five series with 10 sections/each. Spinal nerve ventral roots were harvested at 14 days post RE and cut at 15 µm transversal sections obtaining 3 slices with of 10 slides.

### Immunohistochemistry and image analysis

We treated slides with Tris-buffered saline (TBS), TBS with 0.3% Triton-X-100 and 10% bovine serum for 1 h and incubated overnight at 4 °C with different primary antibodies: goat anti-choline acetyltransferase (ChAT; 1:50; Millipore), chicken anti-Neuro Filament 200 (NF-200; 1:1000, Millipore), mouse anti-Neuro Filament (RT97, 1:200, Hybridoma Bank), rabbit anti-glial fibrillary acidic protein (GFAP; 1:1000, Dako), rabbit anti-ionized calcium binding adaptor molecule 1 (Iba1; 1:1000, Wako), rabbit anti-growth associated protein-43 (GAP43; 1:50, Millipore), mouse anti-chondroitin sulfate proteoglycan (CSPG; 1:100, Hybridoma Bank), mouse anti-Syntaxin 1 (SYT1; 1:100, Hybridoma Bank), rabbit anti-phospho ribosomal protein S6 kinase (Thr 389) (p-pRPS6KB 1; 1:100, Antibodies Online), and rabbit anti-phospho protein kinase B (Ser473) (pAKT; 1:500, SantaCruz Biotechnology). After several washes with TBS with 0.1% Tween-20, the sections were incubated 1.5 h at room temperature with the appropriate Cy-3, Cy-2, or Alexa 647 conjugated secondary antibodies (Jackson Immunoresearch, West Grove, PA, USA). After TBS with 0.3% Triton-X-100 and TBS, Ce counterstained the sections with DAPI (Sigma, St Louis, MO, USA) and NeuroTrace Fluorescent Nissl Stain (Molecular Probes, Leiden, Netherlands) and mounted the slices with Fluoromount-G mounting medium (SouthernBiotech). Immunolabeling of different groups to be compared, and image captured were performed the same day.

We acquired images under the same exposure, sensitivity, and resolution from spinal cord samples of the different treatments or controls for each marker (n = 4/group). Images were captured with the aid of a digital camera (Olympus DP76) attached to the microscope (Olympus BX51) and analyzed with ImageJ software (National Institutes of Health; available at http://rsb.info.nih.gov/ij/). We transformed the microphotographs to a gray scale and analyzed immunoreactivity by calculating the integrated density of a region of interest (ROI) after defining a threshold for background correction.

Glial reactivity was measured on eight spinal cord sections (separated by 200 μm between pairs) immunolabeled against GFAP and Iba1 per animal. Images were taken at 20× and the integrated density of a ROI of 0.11 mm^2^ selected on the gray matter of the ventral horn was calculated. CSPG and GAP43 immunoreactivity was assessed after image capturing at 20× within the white matter located in front of the ventral horn. For both markers, a ROI of 0.11 mm^2^ was used.

To count the number of ChAT^+^ or GAP43^+^ fibers in the nerve sections, images at 10X were taken of one series of eight sections at midlevel of sciatic nerve. For both markers, fibers were counted as positive when co-localization between Neurofilament and ChAT or GAP43 was found.

Confocal microscope examinations were made with a Confocal Laser Scanning Microscope (Zeiss LSM 700) for p-RPS6KB1 and pAKT. Images were collected with a 1.4 numerical aperture oil-immersion 20 or 40X objective. Confocal images were obtained using two separate photomultiplier channels, either concurrently or in separate runs, and were separately projected and merged using a pseudocolor display showing green for Nissl, red for Cy-3, and blue for DAPI. MN area was taken using Nissl green as the ROI, and the integrated density was obtained for at least 15 MN per animal.

### Motor neuron counting

Six slices covering all the L4-L5 segment (separated by 100-µm; n = 4 per group) of eight sections of every animal were incubated 20 min with fluorescence Nissl labeling solution (Life Technologies) following the manufacturer’s protocol. We took sequential microphotographs covering ventral horn (L4 and L5 segments) at 10X with a digital camera (Olympus DP76) attached to a microscope (Olympus BX51). Only MNs localized in the lateral ventral horn with prominent nuclei and soma diameter larger than 30 µm were counted. MN survival was calculated as the percentage of the number of surviving MNs on the ipsilateral side with respect to the contralateral non-injured side of each animal. For retrogradely labeled MNs, the same sections used for MN survival were observed under fluorescence, and the number of labeled neurons counted in every fifth section following the fractionator principle^46^.

### Neuromuscular junction analysis

Gastrocnemius muscle longitudinal slices were washed with TBS, blocked with NDS during 1 h and incubated overnight at 4 °C with chicken anti-Neuro Filament 200 (NF-200; 1:1000, Millipore) as described above. After after several washes, Cy3-conjugated secondary antibody was added. Finally, we washed and incubated slices with FITC-conjugated α-bungarotoxin labeling solution (Life Technologies) to reveal the motor end plates during 20 minutes. Systematic analyses were performed through three different sections of each animal. Bungarotoxin positive endplates (100) were classified as positive and negative for NF-200. Only motor endplates with NF-200 co-labeling were counted as reinnervated per animal (n = 4 animals per group).

### Muscle fiber area analysis

Transversal and longitudinal gastrocnemius muscle sections were stained with H&E. Briefly, nuclei were stained with Harris hematoxylin for 6 minutes followed by differentiation with acid solution of 0.01% HCl in ethanol. Cytoplasm was stained with eosin for 1 minute. Sections were dehydrated by graded ethanol (50%, 70%, 96%, and 100%, and glycerol; twice, 5 minutes each solution) and mounted with DPX. We randomly took images under light microscopy at 20× or 40×, and the areas of at least 100 muscle fibers from three different images were calculated. The mean and area distribution for each animal was analyzed (n = 4 per group).

### Statistical analysis

All values are presented as means ± standard error of the mean (SEM). Statistical analyses were performed using GraphPad Prism 5 software by unpaired t-tests or two or one-way analysis of variance (ANOVA) followed by Bonferroni’s multiple comparison tests. CMAP apparition was analyzed using the Mantel-Cox test, being each CMAP an event. A p value of 0.05 was taken to indicate significant difference.

### Data Availability

The datasets generated during and/or analysed during the current study are available from the corresponding author on reasonable request.
